# Generative AI's Impact on the Mental Health of Medical Students: Scenario Analysis

**DOI:** 10.2196/85373

**Published:** 2026-05-26

**Authors:** Nora Arvai, Bertalan Meskó, Gellért Katonai

**Affiliations:** 1 Kálmán Laki Doctoral School of Biomedical and Clinical Sciences, University of Debrecen, Egyetem tér 1, Debrecen, 4032, Hungary, 3652258058

**Keywords:** mental health, generative AI, generative artificial intelligence, medical curriculum, medical education

## Abstract

**Background:**

Generative artificial intelligence (AI) is quickly changing medical education, even as medical students still face high levels of stress, anxiety, and burnout. These simultaneous trends—technological upheaval and ongoing mental health issues—bring up important questions about how future doctors will be trained and supported. Understanding how these factors might influence each other is crucial for developing resilient, future-ready medical education systems.

**Objective:**

We carried out a foresight study using scenario analysis to examine potential futures at the crossroads of generative AI adoption and medical students’ mental health. An initial environmental scan of the literature was conducted to pinpoint emerging trends and weak signals related to AI in medical education and well-being. These phenomena were categorized within a macro-meso-micro framework and analyzed through a multilevel sociotechnical change perspective. The study focused on 2 principal factors: the extent of generative AI integration into medical curricula and the availability of mental health support, as key drivers and critical uncertainties influencing future trajectories.

**Methods:**

These dimensions resulted in 4 distinct scenarios: Analog Happiness (high support and low AI integration), Gen AI Paradise (high support and high integration), Disconnected Struggles (low support and low integration), and Gen AI Takeover (low support and high integration). Each scenario demonstrates how various institutional responses can impact students’ digital readiness, psychological well-being, and professional growth. For each one, we identified the main systemic risks and suggested immediate institutional measures to address them.

**Results:**

The findings suggest that technological innovation and mental health support must coevolve in medical education. Prioritizing one without the other risks producing either digitally unprepared or emotionally fragile physicians. Faculty readiness, ethical frameworks, and participatory curriculum design are critical to ensuring balanced integration. We formulated practical recommendations tailored to students, educators, and other stakeholders to guide balanced adaptation.

**Conclusions:**

Generative AI is more than just an additional tool in medical education; it is a systemic force that redefines how future physicians learn and operate. If technological change and student mental health are tackled separately, medical education risks creating graduates who are either unprepared for digital demands or mentally overwhelmed. This study highlights key systemic risks and suggests initial institutional steps to address them, providing a foresight-driven framework to assist educators and policymakers in responsible AI integration while safeguarding the well-being of future doctors.

## Introduction

At the beginning of the 21st century, the field of health care (HC) started to experience a cultural shift known as digital health. Digital health can be defined as the cultural transformation that occurs when disruptive technologies provide digital and objective data, accessible to both caregivers and patients, leading to an equal level of doctor*-*patient relationship with shared decision*-*making and the democratization of care [1,2].

Numerous studies have shown that the mental well-being of health care professionals (HCPs) and medical students (MSs) is poor, with high levels of stress, anxiety, and burnout being commonly reported [2-5], even though their welfare would increase the quality of care, productivity, and patient satisfaction [6]. Burnout usually increases gradually, with the highest levels typically occurring during the fourth year of medical school [7].

Studies suggest that artificial intelligence (AI) could bring relief to both the professional duties and academic challenges faced by HCPs and MSs by offering support in diagnostics, decision-making, data analysis, especially administrative tasks, improved consultations, personalized health guidance, medical record analysis, precision medicine, and custom treatment plans [8].

Among various AI technologies, generative AI stands out due to its potential impact on the work of HCPs. Generative AI is the first form of AI to be used by hundreds of millions of people on a daily basis. It has attracted the attention of more than 100 million users in only 2 months [9].

The spread of generative AI has had a particularly significant impact on medical training, as it has fundamentally changed students’ learning habits and called into question the legitimacy of traditional assessment methods [10-18]. MSs’ experience these effects in diverse and highly individual ways.

Generative AI, represented by large language models (LLMs), has the potential to transform HC and medical education. In particular, the impact of generative AI on higher education can alter both students’ learning experiences and faculty members’ teaching practices [19].

Generative AI tools, especially LLMs such as ChatGPT (OpenAI), are designed to create new content from their trained parameters. With free access online and an easy-to-use conversational interface, ChatGPT quickly accumulated more than 100 million users within the first few months of its launch [20,21]. New opportunities for learning, assessment, and research in medical education have sparked excitement, alongside concerns about the implications of cheating and plagiarism in assessments [15]. Studies showed that these models excelled in many areas of medicine previously thought to be exclusive to humans, such as performing well on the United States Medical Licensing Examination, taking a patient history to build a differential diagnosis, diagnosing complex cases, and accurately answering patient questions. These preliminary investigations have shown that generative AI systems have the potential to be generalizable throughout many aspects of medical education, clinical decision-making, and the HC workflow [16]. Concerns have been raised about ethical considerations and the decreased reliability of the existing examinations. Furthermore, in medical education, curriculum reform is required to adapt to the revolutionary changes brought about by the integration of generative AI into medical practice and research [19,22].

As future HCPs prepare for this new reality, they are already incorporating generative AI tools into their learning, which presents challenges for both students and educators, and it seems MSs show curiosity and openness to participate in training related to this field [22].

Both MSs and educators now require skills and competencies that were previously unnecessary. As more patients and HPs use AI-based tools, LLMs being the most popular representatives of that group, it seems inevitable to address the challenge of improving this skill [23]. How stakeholders will respond to this new situation remains to be seen.

The gap between what technology could do and what technology is actually being used for is rapidly widening. While many solutions are proposed to address this gap, clinician resistance to the adoption of AI remains high [24].

We have already demonstrated that although AI can support HCPs in numerous ways, many of them still approach it with fear and perceive AI as a potential threat rather than an opportunity. Their concerns include fear of job displacement, replacement, erosion of professional identity, and loss of expertise, losing control to AI, leading to deskilling and overreliance on technology, and they also dread the potential negative impact on doctor-patient relationships. It seems that the key to helping them successfully implement medical AI into their everyday practice is to address HCPs’ and MSs’ concerns, which requires comprehensive training, ethical guidelines, and ongoing support [25].

With this study, we aim to explore possible futures of medical education with a particular focus on the mental health of MSs. We examine how the mental well-being of MSs may be affected by changes resulting from the integration of AI into medical education. We do that in order to help stakeholders successfully adapt to these changes and effectively tackle the new challenges they face. Instead of predicting a single outcome, we explore how different tech and support setups influence education and career growth. As a foresight study, we do not test hypotheses; instead, we build multiple futures to reduce bias and guide strategy. To assess this, we apply a futures research method, scenario analysis (SA), a well-known method in foresight. It helps to discover possible future scenarios based on key driving forces and major uncertainties.

## Methods

### Overview

This study uses an exploratory foresight design based on SA instead of predictive modeling. SA was chosen because rapid generative AI development and complex mental health dynamics make linear forecasting unsuitable. It identifies key drivers and uncertainties to create coherent, plausible futures that aid strategic reflection rather than prediction.

### Exploratory Scanning

This study used a qualitative foresight methodology to explore the potential futures of medical education, with a focus on MSs’ mental health and the integration of generative AI. SA is rooted in the structured construction of unique and complex scenarios, and allows organizations to build coherent narratives about possible developments. A scenario is a “story” or a narrative illustrating visions of possible futures.

This approach diverges from the concept of a single “most likely future” and instead considers multiple alternative futures.

The scenario approach, originating from the idea of a “script,” can be likened to versions of literary works adapted for stage or screen. Much like a repertory theater, where it is unclear which play will be performed, we prepare for multiple possibilities and interpret the situation from subtle cues to determine which one is unfolding. In essence, scenario building is a way of rehearsing the future in advance. For scenarios to be effective, they must be believable, coherent and logical, and insightful about the future, so they can contribute to decision-making [26].

**Figure 1.**
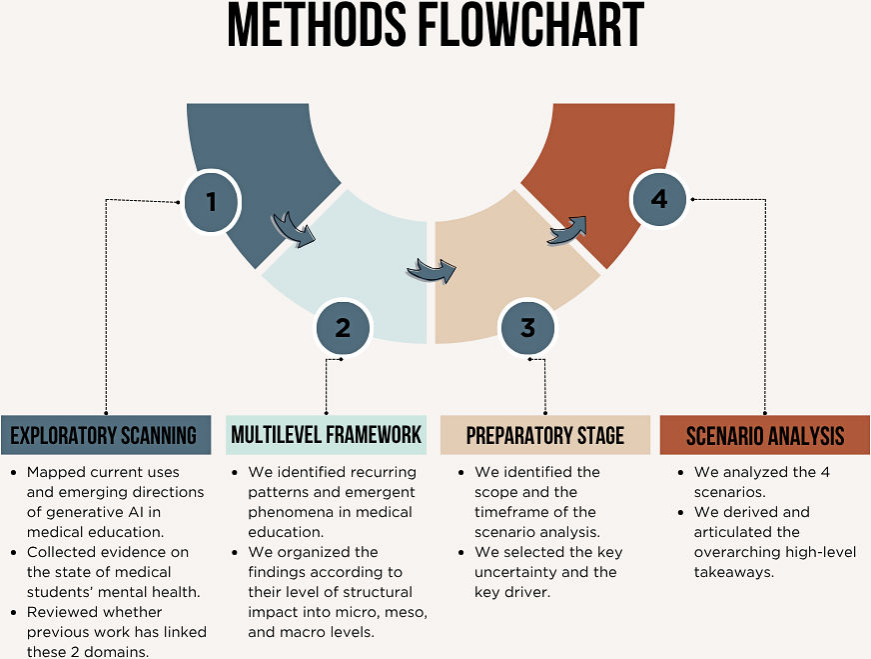
Flowchart of the scenario analysis design used to examine the future of medical education. AI: artificial intelligence.

The entire methodology process is shown in Figure 1 below. The figure outlines the methodological steps of this foresight study, including environmental scanning, identifying key phenomena impacting medical students’ mental health and generative AI integration, selecting critical uncertainties, and developing 4 future-oriented scenarios.

The research process began with structured exploratory scanning, informed by targeted searches of the PubMed database.

This exploratory scanning phase was not designed as a scoping or systematic review, but as a literature-informed environmental scan consistent with foresight and horizon-scanning methodology. Environmental scanning is the process of systematically exploring and interpreting relevant external information to identify potential opportunities, threats, trends, and emerging issues that may affect future planning or decision-making [27]. Environmental scanning is widely applied in foresight research and HC strategy, especially when aiming to identify weak signals and emerging trends. Weak signals are ambiguous early indicators of possible but not certain changes that may become significant in the future [28].

Emerging trends, in contrast, are patterns of change that are becoming increasingly visible and coherent across time and domains. They have more support in the present and suggest a directional shift that is gaining momentum [29].

Environmental scanning provides a broader lens than formal reviews, making it well-suited for the exploratory nature of SA. For example, the World Health Organization’s Global Health Foresight program uses environmental scanning to identify early indicators of potential health system disruptions.

To effectively navigate the existing literature, we formulated the following 3 guiding questions: What are the main applications and emerging directions of generative AI in medical education? What do we know about the global state of MS’s mental health? And finally, have any studies previously examined the intersection of these 2 areas?

PubMed was used as an authoritative entry point to identify relevant peer-reviewed signals and recurring patterns, rather than to achieve exhaustive coverage or formal evidence synthesis. From these recurring patterns, we identified a set of emergent phenomena that illustrate early signs of transformation at the intersection of generative AI and student mental health in medical education. The identification and clustering of these phenomena followed a macro-meso-micro framework adapted from established conceptual models in health systems research [30]. This allowed us to organize the findings according to their level of structural impact, from broad systemic trends to institutional dynamics and individual-level experiences. Therefore, no formal inclusion or exclusion criteria, study appraisal, or qualitative coding procedures were used, since the goal was to understand and construct the scenario rather than to classify evidence.

To ensure analytical robustness, we only selected phenomena that were supported by at least 3 independent and credible sources. In this study, we define phenomena as early, identifiable patterns and dynamics within medical education that reflect the emerging influence of generative AI. These are not merely isolated observations, but conceptually meaningful trends that have appeared across multiple credible sources.

Drawing on the logic of environmental scanning and futures studies, these phenomena represent early indicators, known as “weak signals,” of potential systemic transformation. Each selected phenomenon points to an area where change is already underway and may accelerate.

By defining and clustering these phenomena, we aim to build plausible scenario narratives and anticipate future needs, risks, and opportunities. In total, we identified 5 macro-level, 5 meso-level, and 7 micro-level phenomena, which were categorized and analyzed based on their thematic focus and level of impact (macro, meso, or micro). For this, we used a multilevel conceptual framework. The identified phenomena, along with their brief descriptions and supporting references, are presented in Multimedia Appendix 1.

### Multilevel Framework

A conceptual framework is a network of interlinked concepts that provides a comprehensive understanding of a phenomenon or problem by organizing and structuring related ideas and relationships in a coherent way [30]. Several studies in HC previously used this conceptual framework and have supported it with a micro-, meso-, and macro-level analysis, so we followed the same path [31-33].

To further structure and interpret the identified phenomena, we applied a multilevel framework, which conceptualizes sociotechnical change across 3 analytical levels: macro (landscape), meso (regime), and micro (niche). This approach draws on the foundational work of Geels and Schot: the typology of transition pathways [34], the articulation of multilevel dynamics [35], and a critical review and theoretical refinement of the multilevel framework [36]. Representative examples of phenomena identified at each analytical level illustrate the breadth of the scanning process. On a macro scale, broad digital transformation, the swift spread of generative AI, and the global mental health crisis in medical training emerged as key structural influences. At the meso level, inconsistent integration of AI curricula, faculty resistance or adaptation, and differences in institutional mental health support reflected diverse organizational reactions. At the micro level, factors such as AI-related technostress, concerns about academic integrity, and hesitance to seek psychological help showed how systemic and institutional challenges impacted individual experiences.

Not all these phenomena became core dimensions; only those with high impact and variability across different settings were combined into the 2 main dimensions used for scenario development.

### Preparatory Stage and Scenario Analysis

This approach allowed us to map the landscape of relevant research with clarity and accountability, serving as a robust foundation for subsequent SA. This structured and multivalidated selection process provided a strong foundation for identifying key trends, driving forces, and critical uncertainties used in our SA [37]. To create a detailed scenario analysis, we followed 5 well-defined steps.

First, in the preparatory stage, we defined the scope and time horizon. The scope is the transformation of medical education driven by generative AI and the mental health status of students. Given the rapid pace of technological development, we set a time horizon of 5 years.

Second, we identified key drivers and uncertainty. Key drivers are the primary factors or forces that have a significant influence on the outcome of future developments, such as technological advancements, economic trends, societal changes, environmental conditions, and political shifts. Uncertainties are elements or events that are unpredictable and have the potential to significantly impact future outcomes, as the direction or impact is unknown.

We analyzed possible scenarios, and while doing so, we assigned descriptive titles to each of them. We created 4 distinct scenarios by combining the key drivers and uncertainty. After all that, in the fourth step, we analyzed the implications of each scenario and identified opportunities and threats associated with each.

**Figure 2.**
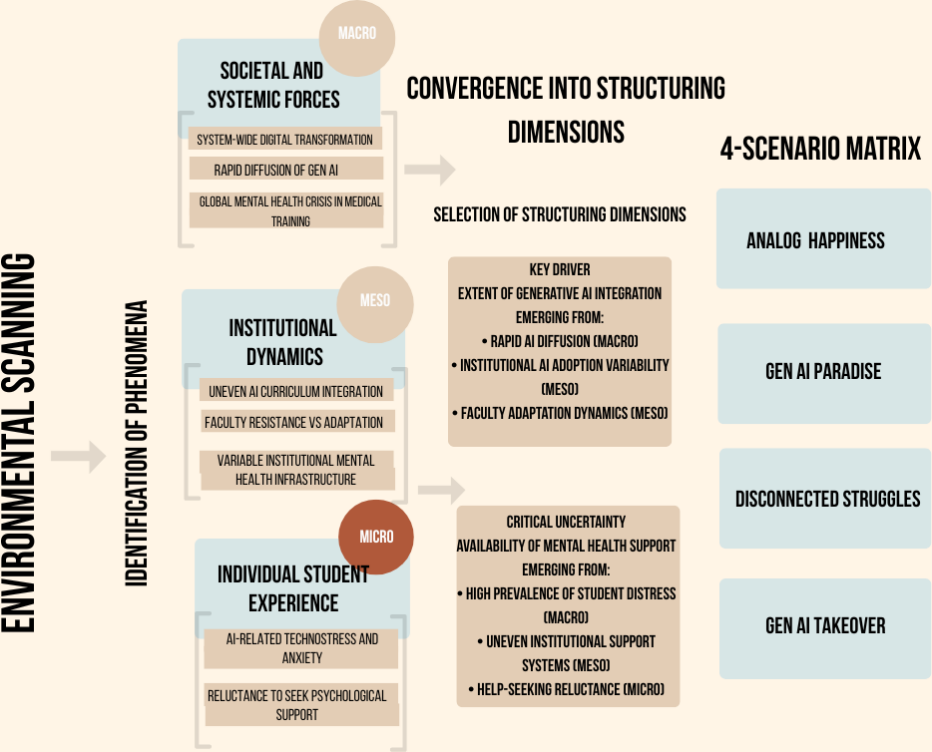
This foresight study on medical education uses an analytical framework for scenario construction. AI: artificial intelligence.

Figure 2 depicts the complete analytical process, demonstrating how exploratory environmental scanning identified phenomena at macro, meso, and micro levels. These findings were then combined into 2 main structuring dimensions: the degree of generative AI integration (a key driver) and the availability of mental health support (a critical uncertainty). This merging laid the groundwork for the 4-scenario matrix. To strengthen plausibility, the selection of the key driver, critical uncertainty, and the resulting scenario structure were reviewed by 2 subject matter experts in foresight and digital health.

### Ethical Considerations

This study did not involve human participants or personal data, so no ethics approval was needed. It used a qualitative review of publicly available literature from PubMed, with all sources properly cited and ethically compliant. The study adheres to the Declaration of Helsinki and JMIR publication standards.

## Results

### Exploratory Scanning

Out of the 3 guiding questions we formulated in the Introduction section, the results for our first guiding question regarding AI applications in medical education reflect a well-researched and rapidly evolving field. Yet, most papers highlight the need for further research to explore the effectiveness of generative AI applications in medical education [13].

Generative AI is increasingly used to support MSs’ learning journeys, providing personalized experiences and improved outcomes.

Generative AI, such as ChatGPT, is accessible and transforming medical education [38]. Boscardin et al [15] propose an AI literacy framework to guide educators in integrating AI into admissions, learning, assessment, and research, helping them adapt to this new environment. As generative AI tools continue to expand, educators need to increase their AI literacy through education and vigilance regarding new advances in the technology, serving as stewards and coaches of generative AI literacy to foster social responsibility and ethical awareness around AI use.

Preiksaitis and Rose [14] conducted a scoping review; their thematic analysis identified diverse AI applications in medical education, such as self-directed learning, simulations, and writing help. They highlighted challenges such as academic integrity, data accuracy, and learning detriments. They proposed 3 research areas: developing critical evaluation skills, rethinking assessments, and studying human-AI interactions [39].

Chan and Zary [39] identified 3 main uses of generative AI in medical education: learning support (n=32), student assessment (n=4), and curriculum review (n=1). Its main benefits are feedback, guided learning, and reduced costs. The subgroup analysis shows that medical undergraduates are the primary audience. Additionally, 34 articles discuss challenges, mainly assessing the effectiveness of AI and technical development issues in applications.

The increasing use of generative AI in medicine has raised ethical concerns, such as patient autonomy, bias, and transparency. Many of the recent studies suggest a need for teaching AI ethics as part of medical curricula [40].

The second guiding question we asked was what we know about students’ global mental health. This also proved to be an area of strong scientific interest.

Nair et al [3] found that students’ mental health concerns manifest in various forms. MSs facing academic pressure, debt, sleep deprivation, illness exposure, and mistreatment may explain the higher rates of disorders. These difficulties are dynamic, with certain challenges becoming more prominent during key student transitions in education. Primary examples include the entry year of medical school, the shift from preclinical curriculum to clinical training, and the final moments prior to beginning residency. The authors concluded that COVID-19 worsened stress, anxiety, and depression in medical education. They highlight solutions such as crisis management training, peer support, destigmatizing mental health, and better access to resources, emphasizing that it is up to medical education leaders to implement them.

Sleep deprivation is a major concern, with many medical students experiencing sleep problems that affect their academic performance and mental health. Perotta et al’s [41] study found that students with more sleep deprivation had higher odds of anxiety and depression and lower odds of good quality of life or positive educational perception environment.

According to Backović et al [4], medical studies bring many stressful activities to students. Prolonged stress can have adverse effects on mental health and lead to further professional burnout. Studies across different countries have consistently demonstrated high rates of several psychiatric disorders among MSs.

Studies suggest that mental health worsens after students begin medical school and remains poor throughout training. On a personal level, this distress can contribute to substance abuse, broken relationships, suicide, and attrition from the profession. On a professional level, studies suggest that student distress contributes to cynicism and may subsequently affect students’ care of patients, relationships with faculty, and ultimately the culture of the medical profession [42].

According to Watson et al [43], MSs’ suicide likely relates to social, environmental, and endemic factors such as curricula, accommodations, support, pressures, social isolation, competition, early separation, simulation, cadavers, and trauma in training. The sociocultural environment, including ragging, expectations from teachers and patients, may pressure vulnerable students. Higher rates among females raise questions about gender roles and expectations in medicine.

Worldwide, medical schools have responsibilities to respond to concerns about students’ psychological, social, and physical well-being, but guidance for medical schools is still limited [44]. Depression among MSs may be undertreated. Medical schools can assist depressed students by addressing issues such as the stigma of using mental health services, confidentiality, and documentation. Early treatment of impaired future caregivers may have far-reaching implications for the individual students, their colleagues, and their future patients [45].

Despite potential issues, MSs often avoid professional help to protect their identity. They believe medical students should be invulnerable. Hankir et al [5] note that MSs and doctors rarely seek help until a crisis, with stigma fears hindering access to mental health services.

Autobiographical narratives of the “Wounded Healer” are increasingly popular among MSs and doctors with mental health challenges, serving as both adjunctive therapy and antistigma campaigns. A randomized controlled trial of Coming Out Proud with mental illness showed immediate positive effects on stigma and stress variables [46].

Dederichs et al [22] highlight that many students with mental health issues do not seek help, creating a need for easily accessible interventions. Several evidence-based internet- and mobile-based interventions (IMIs) have been suggested, and researchers are interested in students’ views on them. Students appreciated IMIs for their easy access, flexibility, ability to reduce waiting times, and as an initial step before in-person therapy. Nonetheless, medical students favored face-to-face treatment for serious mental health issues. Their findings show generally positive attitudes toward IMIs for mental health promotion, though concerns remain about their effectiveness for severe disorders and emergencies. These results suggest that IMIs could be valuable tools for stress prevention and early intervention among students. It is also noteworthy that students specifically preferred certified IMIs recommended and provided by trusted sources such as universities.

The growing presence of medical influencers on platforms such as TikTok (ByteDance) and Instagram (Meta Platforms, Inc) has introduced a new kind of voice into conversations around mental health in medical education. Although they are not necessarily tied to formal institutional structures, they are still part of the professional community, speaking both to the public and to insiders, such as MSs and residents.

Their content extends beyond personal stories to shaping norms. It addresses mental health training, maintaining well-being, and institutional expectations, influencing students’ views on education and their future profession. In this way, they contribute indirectly to the broader culture and practices of medical institutions. Their influence on individuals is significant, resonating emotionally with students through messages such as “you’re not alone” or “I’ve been through this too,” which offer support and practical advice. Many find this more authentic than official messages. These influencers act as digital boundary spanners, bridging the gap between the system and people, operating between institutional and personal levels. While writing this, Jake Goodman boasts 1.2 million followers on TikTok; his top video has reached 2.1 million viewers. He has spoken at the White House and given a TED Talk. He publicly addresses the mental health crisis in medical training, emphasizing the high depression rates among students, the ongoing 24-hour shifts, and how the fear of retaliation discourages open discussion and meaningful reforms.

On Instagram, numerous coaches present themselves as business mentors, specifically teaching HCPs how to avoid burnout by working part-time and building a side hustle. Numerous influencers similarly focus on helping HCPs create and sell online courses, also as a strategy to reduce workload, increase income, and prevent burnout.

This phenomenon is gaining acceptance and offers an alternative for HCPs seeking balance and sustainability. However, the profession faces a severe workforce shortage, increasing pressure on those in clinical roles. The system must train and retain more physicians or make the profession more appealing by creating humane working conditions and restoring purpose and dignity, starting from medical training. Medical AI offers a promising opportunity in this regard, as discussed in our previous article [25].

The result of searching for the third guiding question—the intersection of the previous 2 topics—does not appear to be a particularly well-explored area. Its investigation remains largely open, offering an opportunity for original contribution. It is, however, worth noting a few preliminary mentions in the literature.

The findings of Li and Quin [11] show that medical postgraduate students are more aware of generative AI than undergraduates. Their intention to use it links to performance expectancy, habit, hedonic motivation, and trust. Medical education should focus on enhancing training performance with courses that are easy and engaging to equip students [11].

Thesen et al [47] introduced a precision well-being framework for medical education, based on a comprehensive view of mental health, combining mental health and positive psychology measures in a data-driven way. Using unsupervised machine learning on data from 3632 MSs, clusters were formed based on depression, anxiety, and flourishing metrics. Three distinct clusters were identified. Membership in the “Healthy Flourishers” well-being phenotype was associated with no signs of anxiety or depression while simultaneously reporting high levels of flourishing. Students in the “Getting By” cluster reported mild anxiety and depression and diminished flourishing. Membership in the “At-Risk” cluster was associated with high anxiety and depression, languishing, and increased suicidality. Nearly half (49%) of the medical students surveyed were classified as “Healthy Flourishers,” whereas 36% were grouped into the “Getting-By” cluster and 15% were identified as “At-Risk.” Findings show that a substantial portion of MSs report diminished well-being during their studies, with a significant number struggling with MH challenges.

Thesen et al’s [47] precision well-being framework is an integrated model that classifies medical students into well-being phenotypes based on their mental health. It supports student aid and evaluates personalized interventions, illustrating the role of AI in assisting MS.

While reviewing the literature, we found only 2 studies that use SA to explore the future of medical education, and one that used scenario planning. The first study used ChatGPT to develop the scenarios, the second examined the future of midwifery practice in Antwerp, and the third aimed to explore the potential of scenario planning to bridge the understanding gap and frame strategic planning for interprofessional education and practice, as well as to implement innovative techniques and technologies for large-group scenario planning.

Knopp et al [48] used generative AI to create 4 scenarios: AI Harmony, AI Conflict, Ecological Balance, and Existential Risk. Risks include disinformation, privacy loss, inequality, reduced autonomy, and ethical issues. Benefits are efficiency, personalized interventions, better collaboration, early detection, and faster progress in research.

The second study explored the future of midwifery praxis using structuration theory and intuitive logics scenario planning methods to structure contextual midwifery scenarios. Three scenarios described the plausible future of midwifery: (1) midwife-led care monitoring maternal health needs, (2) midwife-led holistic care, and (3) midwife- or general practitioner–led integrated maternity care [49]. In the third study, a full-day scenario planning workshop was held, introducing an innovative methodology to 71 participants from 9 universities, service providers, government, students, and consumer organizations. Outcomes were assessed via statistical and thematic analyses of a mixed methods survey. They concluded that the scenario planning method can be used by tertiary academic institutions as a strategy in developing, implementing, and embedding interprofessional education, and for the enculturation of interprofessional practice in practice settings [50].

### Phenomena Identified

After examining study approaches to SA in medical education, we synthesized findings to identify underlying forces shaping the field. Moving from individual studies to broader patterns, we classified phenomena into micro, meso, and macro levels using a conceptual framework. At the macro level, societal forces such as technological pressures and shifting expectations influence medical education but are beyond individual control, shaping what is seen as possible in the future of training. The meso level is the institutional level where most structural changes happen, focusing on medical faculties and educational systems. It examines how universities respond to generative AI and its impact on students, highlighting institutional resistance or adaptation and how AI influences professional development norms.

In our previous work, we summarized the currently available AI-related courses [25]. An important part of these changes is the growing openness around MH: topics once taboo are now openly discussed, often promoted by physician-influencers on TikTok and Instagram. While macro-level shifts enable this, the institutional embrace belongs to the meso level.

**Figure 3.**
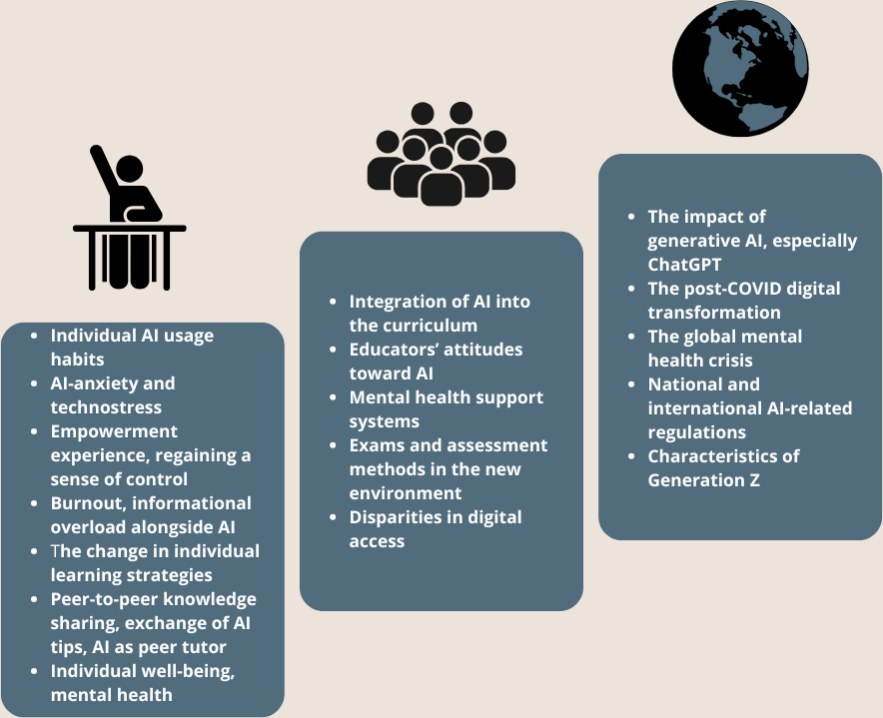
A multilevel conceptual framework is used to categorize factors affecting medical students in this foresight study. AI: artificial intelligence.

At the micro level, we see the most immediate and personal impact of AI, as experienced by individuals. This includes the tools that MSs use in their daily routines, changes in their learning strategies, and, crucially, their mental health. It also involves the coping mechanisms they adopt and how they experience the medical school years on a personal level. A summary table of the phenomena, along with the list of supporting articles, can be found in Multimedia Appendix 1. Figure 3 provides a summary of the phenomena occurring at the 3 levels. The framework identifies micro (individual), meso (institutional), and macro (societal) levels that influence the future of medical education, especially regarding the adoption of generative artificial intelligence and mental health issues. AI: artificial intelligence.

This validated selection process laid a strong foundation for SA, identifying key trends, drivers, and uncertainties. Grounded in current trends and framework phenomena, we analyzed and defined the scope, time frame, key drivers, and main uncertainties to develop the scenarios. All 4 scenarios share a common scope: the transformation of medical education driven by generative AI and the mental health status of students. Given the rapid pace of technological development, we have set a 5-year time horizon, projecting the scenarios to the year 2030.

This time horizon reflects both the rapid evolution of generative AI technologies and the urgency of mental health challenges among MSs. Innovations such as multimodal AI agents, personalized learning platforms, and AI-assisted assessment tools are already being piloted in several institutions, suggesting that large-scale educational disruption is not only possible but imminent [23]. Simultaneously, student mental health has become a widely recognized crisis requiring near-term interventions [2-5,46,51]. Therefore, we chose a 5-year time frame that offers both relevance and feasibility for building the scenarios.

### Driver and Uncertainty Selection

We identified the extent of generative AI integration into curricula as the key driver and access to mental health support as the key uncertainty. These emerged from the multilevel conceptual framework as the phenomena with the most far-reaching impact, best describing and shaping the field.

The key drivers and uncertainties were chosen because they both represent high-impact factors. The first involves technological feasibility, faculty acceptance, ethics, and curriculum redesign. The latter reflects variability in institutional capacity, stigma, funding, and cultural attitudes toward psychological care. Our choices were based on the conceptual framework and supported by at least 3 high-quality sources each, showing their significance as system-wide phenomena that influence.

This methodology serves to map the evolving landscape and provide a coherent foundation for building plausible scenarios. To identify the key driver and critical uncertainty, candidate dimensions were evaluated based on three criteria: (1) their ability to integrate multiple phenomena across macro-meso-micro levels, (2) their structural impact on the future of medical education, and (3) their variability across different institutional settings. Dimensions supported by at least 3 independent, high-quality sources were prioritized. As a result, the level of generative AI integration was selected as the key driver, while the availability of mental health support was identified as the critical uncertainty, reflecting the tension between rapid sociotechnical change and limited psychological support in medical training. This final choice was reviewed by an independent expert to strengthen methodological credibility.

### Scenario Descriptions

#### Scenario 1: Analog Happiness

- Key driver: integration of generative AI into medical curricula is low.
- Uncertainty: access to the MS support system is high.

In the first scenario, there is high access to the MS support system, but the integration of generative AI into medical curricula is low.

We named this scenario “Analog happiness.” Institutions prioritize the mental health of MSs. Inspired by research highlighting the importance of accessible mental health services, universities invest in comprehensive support infrastructures. Mental health is openly discussed, and stigma around it has significantly decreased, influenced by broader cultural shifts and the visibility of physician-influencers advocating for the mental well-being of MSs [52]. There is a strong institutional focus on supporting MSs’ psychological well-being, aiming to address diverse needs through professional and peer-led support, as shown by Hale and Davis [52].

Their study found a peer-led, virtual workshop series effective in reducing stress, anxiety, and depression among MSs. Psychiatrists providing mental health treatment to MSs recognize the importance of managing transference, countertransference, overintellectualization, and occurrences of strong idealization identification [51].

However, the use of generative AI remains marginal. Faculties are hesitant, citing ethical concerns and a lack of guidance [53]. Curricula rely on traditional methods. MSs’ use AI informally, but this use is inconsistent and sometimes ethically ambiguous [21]. Without clear institutional direction, the inappropriate usage of generative AI leads to missed opportunities in personalization and innovation.

Students may be emotionally supported but lack digital readiness. Educational inequities may widen between digitally progressive and resistant institutions. Alkhaaldi et al’s [21] study found that surveyed MSs had little experience with AI tools and limited perceptions of AI in health care, but they generally viewed ChatGPT and AI positively and were optimistic about the future of AI in medical education and HC. With structured curricula and formal policies and guidelines, it would be possible to adequately prepare MSs for the forthcoming integration of AI in medicine, so this scenario is the future of missed opportunities.

#### Scenario 2: Gen AI Paradise

- Key driver: integration of generative AI into medical curricula is high.
- Uncertainty: access to mental health support is high.

In the second scenario, both access to mental health support and integration of generative AI into medical curricula are high. We referred to this scenario as “Gen AI Paradise.” This scenario represents a future in which medical education institutions have both embraced generative AI and prioritized student mental well-being.

AI tools are deeply integrated into all levels of the curriculum, guided by international best practices and ethical standards [19,54]. Students learn to use generative AI tools for research, simulation, and diagnostics, and critically reflect on their implications, building AI literacy.

Educators intentionally integrate AI into curricula based on research such as Shimizu et al [19], analyzing the impact of AI on medical education and suggesting reforms. The authors use SWOT (Strengths, Weaknesses, Opportunities, Threats) analysis and identify 3 main learning approaches: “learning about GenAI,” “learning with GenAI,” and “learning beyond GenAI.” The study emphasizes the importance of teaching the ethical use of generative AI, the benefits of adaptive learning, and the continued value of nonsubstitutable skills such as communication and clinical experience [19]. MSs also use generative AI tools as virtual patient and clinical decision-making tutors [55]. Robust mental health services are tailored to MSs’ psychological needs, differing from those of previous groups. Their design is based on research such as Duan et al [18], highlighting student-centered, stigma-free MH strategies in medical schools. They analyzed MSs’ perceptions, trust, and attitudes toward generative AI in medical education, and explored their willingness to integrate generative AI in learning and teaching practices.

The majority of participants reported familiarity with generative AI concepts, whereas only 43.5% had an understanding of generative AI applications specific to medical education. In this ideal future, decision-makers also take into account age- and gender-related differences, ensuring that support systems and educational strategies are responsive to the diverse needs of students.

Postgraduate students exhibited significantly higher levels of awareness of generative AI tools in medical contexts compared with undergraduate students. Male students were more enthusiastic and engaged with AI than females. Females expressed more concerns about privacy, security, and ethics in AI for medical education. Male or postgraduate students showed stronger intentions to use these tools in future learning and teaching practices [18].

In this scenario, technostress and other generative AI–related anxieties are proactively addressed, and digital well-being is embedded into the culture of medical training. Faculty members are supportive and trained to use generative AI in ways that reduce, rather than increase, student burden. Educators are trained to guide students in ethical, effective generative AI use.

Simultaneously, mental health support is robust and student-centered. Further, generative AI is recognized as a tool that can enhance efficiency and involve patients in education. Stakeholders understand that medical education should focus on competencies that generative AI hardly replaces, such as clinical experience and communication. Notably, involving both faculty and students in curriculum reform discussions fosters a sense of ownership and ensures broader perspectives are encompassed [19].

Technostress is mitigated through proactive digital well-being education [56]. Students report reduced anxiety, increased self-efficacy, and improved learning satisfaction. This scenario reflects a successful co-evolution of technology and care.

This model enhances academic efficiency and personalization while safeguarding emotional health. It may become the benchmark for 21st-century medical education.

#### Scenario 3: Disconnected Struggles

- Key driver: the level of generative AI integration is low.
- Uncertainty: access to mental health support is low.

In the third scenario, access to mental health support and the level of generative AI integration are low. We named this scenario “Disconnected struggles.” In this scenario, institutions fail to adequately respond to either the emotional or technological challenges of contemporary medical education. Mental health services remain limited, fragmented, or stigmatized, even as students face rising levels of stress and burnout [57,58].

The pandemic-induced digital shift exacerbated isolation, yet long-term MH responses have been inconsistent [59,60]. Generative AI tools are largely ignored at the curricular level, with little institutional support and faculty lacking interest or digital fluency. Students attempt to use AI without guidelines, causing uncertainty, inconsistent results, and anxiety [58].

This creates a high-pressure, low-support environment where learners face digital and emotional disorientation. Students’ well-being is declining, while academic inequalities are widening and trust in institutions is eroding. The lack of coordinated support during rapid digital transitions places students at risk of experiencing what might be described as “academic trauma,” marked by confusion, anxiety, and a diminished sense of belonging within the educational environment. There is ambiguity around when students should use these tools, what constitutes plagiarism, and how to detect when text has been authored by generative AI. Most importantly, there is a lack of availability and experimentation, as well as a lack of competencies and guidance on how to incorporate the new and rapidly evolving world of generative AI and LLMs into the core medical school curriculum and experiences of medical education [16].

Even professors are confused, and they seem to be afraid of generative AI. Cervantes et al [61] state that faculty mainly worry about cheating (97%), errors in AI (95%), lack of context (86%), and reduced human feedback (83%). They cite the absence of safe use guidelines from the government and institutions. Key challenges include cheating, AI mistakes, and privacy and safety concerns. Those who themselves fear the technology are neither able to teach it effectively nor represent it credibly, and in this scenario, they do not even intend to.

#### Scenario 4: Gen AI Takeover

- Key driver: the level of generative AI integration is high.
- Uncertainty: access to mental health support is low.

In the final scenario, access to mental health support is low, but the level of generative AI integration is high. The name of this scenario is “Gen AI Takeover.” This future envisions a system where technological innovation outpaces emotional preparedness, highlighting the risks associated with innovation without adequate support. Institutions push AI integration aggressively [62], embedding tools like ChatGPT into every learning domain.

Students gain AI fluency, but emotional burden grows. Both students and faculty are enthusiastic about generative AI in medical education. Students and trainees use AI for learning, clinical reasoning, and operational tasks, while faculty create curricula, assist teaching, and handle grading assessments [16].

Generative AI tools are thoroughly integrated into the curriculum, driven by institutional enthusiasm and a desire for global competitiveness. Students engage with AI-based tutoring, simulation, and assessment systems from their first year, benefiting from individualized feedback and enhanced learning pathways.

Yet, the emotional cost is high. Mental health services have not evolved in parallel, and students report high levels of technostress, AI anxiety, and burnout [63,64]. Without adequate mental health infrastructure, the fast-paced digital transformation leads to emotional fatigue, performance pressure, and an erosion of intrinsic motivation.

Coping strategies differ: some students thrive, others feel overwhelmed, unsupported, or disconnected. They may feel isolated and disoriented, turning to unregulated online tools or peer forums for support. Without structure, these increase stress, raising burnout and dropout rates. Without adequate mental health systems, generative AI–related anxiety and information overload escalate [65-69].

The curriculum emphasizes performance over well-being. The study by Sallam et al [70] shows that high generative AI integration without emotional support leads to increased anxiety, fear, mistrust, and ethical issues among medical students, highlighting the need for curriculum changes. Interventions should improve familiarity and skills with generative AI to reduce concerns and prepare future HCPs for this essential technology effectively. This study also highlights the importance of incorporating ethical discussions into medical courses to address mistrust and concerns about the human-centered aspects of generative AI.

Their study calls for the proactive evolution of medical education to prepare students for new AI-driven HC practices, ensuring that physicians are well-prepared, confident, and ethically informed in their professional interactions with generative AI technologies [70]. We stress that the low psychological support here is not due to missing AI tools, but rather to a lack of support or its nonprioritization. AI is used for performance and productivity. While AI tools such as chatbots exist, evidence of their effectiveness in preventing mental health issues is limited and mixed. Some studies show benefits; others raise concerns about safety and reliance. We do not assume AI alone guarantees better mental health. The scenario shows AI mainly enhancing performance, while mental health support still develops.

**Figure 4.**
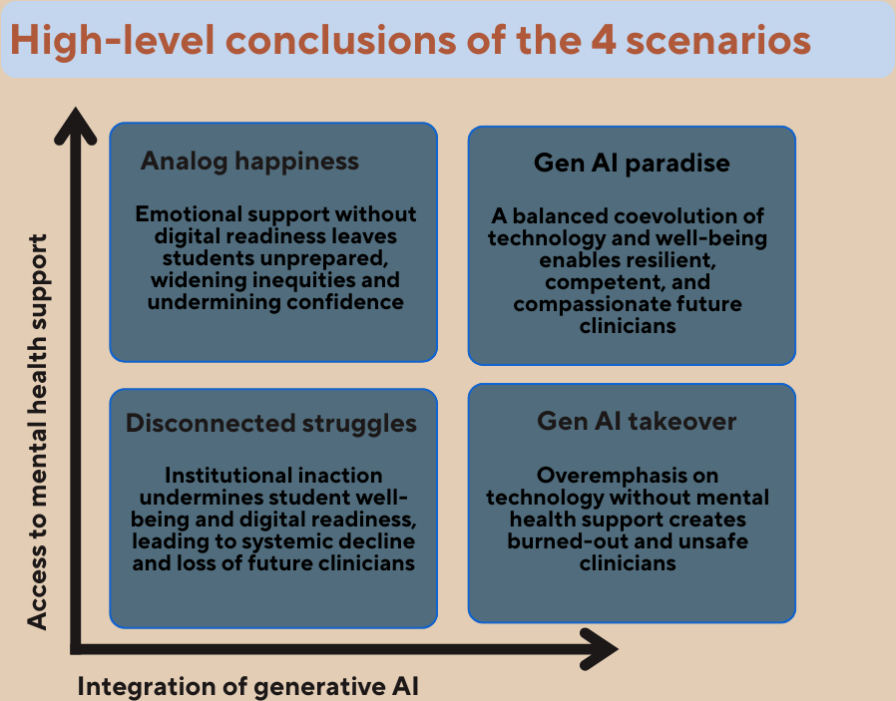
Four-scenario matrix illustrating possible futures of medical education for medical students. AI: artificial intelligence.

In this scenario, the education system is driven by efficiency but devoid of care. Students may become proficient but emotionally fragile practitioners. The 4 scenarios and their high-level takeaways are presented in Figure 4.

### Summary of Key Insights

Over time, the scenarios could be assessed against a set of evolving indicators. On the macro level, shifts in national funding priorities or changes in AI regulation may signal broader structural trajectories. On the meso level, curriculum revisions, the adoption of new pedagogical and exam formats, and institutional data on student well-being can highlight how organizations adapt. At the micro level, longitudinal feedback from students, including patterns of AI use in everyday study practices and self-reported mental health trends, may reveal which scenario resonates most closely with the lived reality in the timeframe of our scope.

## Discussion

### Principal Findings

This study used scenario analysis to explore future medical education, focusing on AI integration and mental health support. To grasp the role of AI, we first examine the current landscape. Medical education faces 2 core challenges: training students to collaborate with generative AI and protecting their mental health, both crucial for learning and developing as clinicians. Our findings build on existing research that highlights the rising use of generative AI and mental health issues among MSs, while also emphasizing the often-overlooked connection between these areas.

The 4 scenarios depict potential futures of medical education based on institutions’ responses to integrating generative AI and MSs’ mental health. Each explores different balances, but the key insight remains: technological innovation must be paired with emotional support. Both are interdependent in developing resilient, future-ready HCPs. The “Gen AI Paradise” scenario illustrates the potential of a balanced future, where AI supports learning and efficiency without compromising the psychological well-being of MSs.

The scenarios highlight risks of unbalanced progress: rapid digital growth without mental health support or stagnation in both areas. “Analog Happiness” shows that even supportive environments fail if MSs are not prepared for digital demands. They reveal that technological innovation and emotional support are interconnected in developing resilient, future-ready HCPs. The “Gen AI Paradise” scenario envisions a future where technological fluency is paired with emotional support and well-being. It exemplifies coevolution, with generative AI enhancing learning and diagnostic skills, while mental health services buffer training stress. AI is seen as a complementary, empowering tool, not a replacement. Institutions invest in infrastructure and culture to ensure students feel supported and involved in shaping their educational paths. This scenario offers a promising foundation for modern medical education, one that does not force a choice between care and competence, but weaves them together. This scenario serves as a call to restore the original contract of medicine, not as a choice between compassion and competence, but as a new lineage of healers who embody both. Modern medical education must remember what we already know, ever since Balint: the doctor is the medicine [71].

Healing is undoubtedly a profession and a scientific discipline; however, it extends beyond that, encompassing qualities such as compassion, empathy, and dedication. It can also be regarded as a calling. And the well-being of the healer is a key driver of both patient satisfaction and recovery [6]. The “Gen AI Takeover” warns of a technocratic future valuing efficiency over MS and educators’ well-being, risking burnout, anxiety, technostress, and disengagement. It highlights societal risks of digital acceleration, lacking human focus, which could weaken future practitioners’ emotional resilience and impact patient care.

The “Disconnected Struggles” scenario depicts institutional paralysis where digital and emotional challenges are ignored. MSs face a high-pressure environment with little support; the absence of AI and mental health resources causes stress, anxiety, mistrust, and trauma. Trauma may result in dropping out, career changes, low self-worth, self-doubt, or suicide. It highlights warning signs such as burnout, unprepared faculty, and ethical disputes in some institutions.

Meanwhile, the “Analog Happiness” scenario shows that a caring educational culture alone is insufficient. Without digital readiness and AI literacy, even the best systems fall behind. Mental health services cannot relieve the tension from unmet needs; they merely chase symptoms, fighting a losing battle. MSs in this scenario are emotionally supported but digitally vulnerable, widening institutional inequities and leaving graduates unprepared for the technological realities of modern clinical practice. There is a growing concern that MSs who are dissatisfied with the current level of generative AI integration will seek out training independently, outside of validated or regulated environments. This increases the risk of misinformation and errors, which may lead to guilt, despair, and a decline in their confidence and belief in their professional abilities.

Across all futures, a key insight is that technological preparedness must grow alongside emotional support systems. Focusing on one creates an unsustainable training environment. Institutions should see generative AI as a transformative force that reshapes learning, decision-making, professional identity, and the doctor-patient relationship. At the same time, the emotional landscape of medical education is changing, with MSs facing increased academic pressure and digital challenges overload. Failing to respond to these dual pressures risks creating emotionally exhausted, uncertain, or digitally unprepared physicians.

Additionally, the scenarios emphasize the importance of faculty readiness and institutional clarity. Without proper training and policy frameworks, even the most promising technologies may create confusion or misuse. Educators themselves must be emotionally equipped and technologically literate to guide students through this new landscape.

The scenarios emphasize the need for inclusive, participatory curriculum reform by involving students and faculty in integrating generative AI and mental health strategies to ensure relevance. Future policies should explore hybrid approaches combining foresight.

This study offers 3 contributions: applying structured scenario analysis to the understudied intersection of generative AI and medical students’ mental health, separating technological integration from psychological support, and using a macro-meso-micro perspective to link systemic changes with individual experience.

### Limitations

This study has limitations. First, scenario analysis is exploratory, not predictive; the scenarios are structured narratives, not forecasts. They aim to stimulate decision-making but cannot capture all future complexities.

Second, the authors mainly developed the scenarios, risking subjective bias despite independent review. Scenario development inevitably involves interpretive judgment. Although a multidisciplinary research team and external expert review helped mitigate bias, alternative drivers or uncertainties could be identified by other researchers.

Third, the scenarios were developed through desk-based, literature-informed synthesis without participatory foresight workshops, which may limit the range of stakeholder perspectives represented. The literature base we used, although broad, is still limited by language, as we relied solely on English sources, and by the rapidly evolving nature of research on both generative AI and student mental health. At the time of our data collection, some weak signals may not yet be visible in the literature or may have emerged later.

Fourth, we set a 5-year horizon for relevance, but this limits exploration of longer-term systemic changes such as cultural shifts in medical education or health system restructuring.

Fifth, we highlighted generative AI and mental health as central dimensions, but other key drivers, such as global health inequities, climate change, financing models, and interprofessional education, were not included in our scenarios. Future research could expand to these systemic drivers.

Our study focuses on medical education in higher-income countries with better digital infrastructure and mental health access. Findings should be cautiously applied to low- and middle-income countries, where conditions differ.

### Conclusion

**Figure 5.**
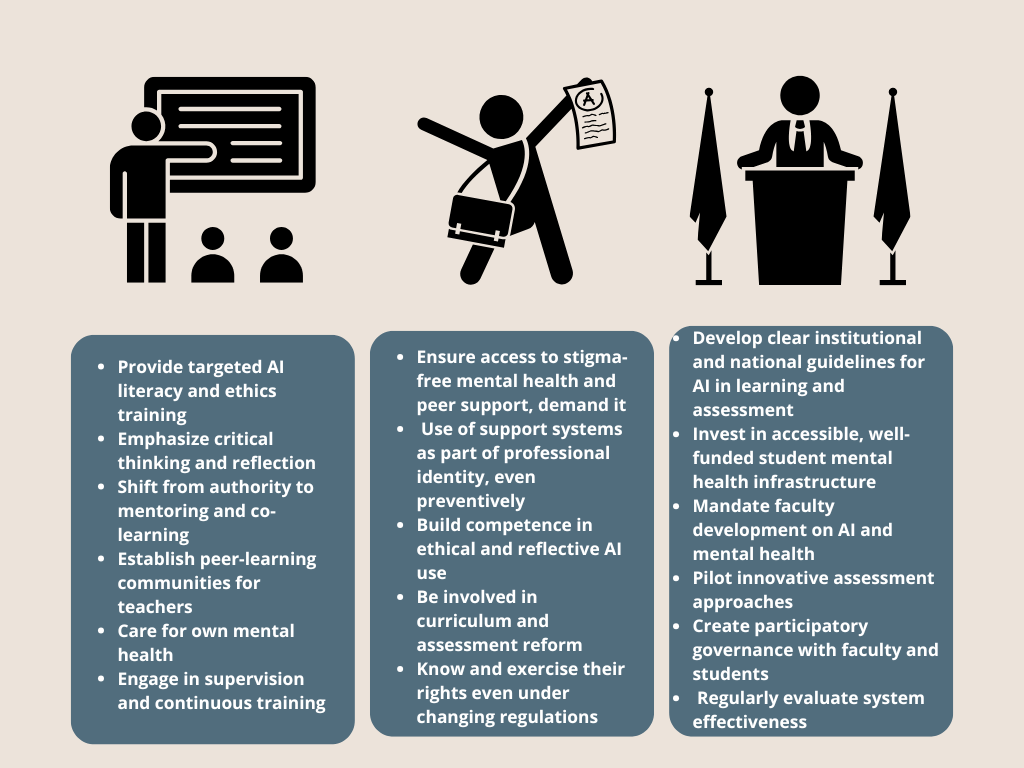
Practical Implications of the 4 future scenarios for key stakeholders in medical education are discussed. AI: artificial intelligence.

Medical education seems to be at a crossroads. Institutions should not see technological and emotional development as competing priorities, but as mutually important pillars of responsible and future-ready education. In Figure 5, we present our practical advice to students, educators, and stakeholders based on different levels of generative AI adoption and mental health support, highlighting potential impacts and strategic considerations for medical students, educators, and institutional leaders.

Ensuring ethical AI use and investing in accessible, stigma-free mental health services are essential. To prepare future HCPs, stakeholders should develop both care and innovation, helping students become digitally fluent and emotionally resilient. Ignoring either the psychological or digital readiness of future HCPs is not a neutral act; it has ethical and professional implications. Institutions must fulfill this dual responsibility, or they risk fostering burnout rather than excellence. This is in the best interests of all stakeholders, educators, MSs, HCPs, and patients, as the mental well-being and technological preparedness of HCPs are critical determinants of clinical performance and, consequently, patient outcomes [6].

Speaking of educators, while the scenarios were student-centered, generative AI also transforms faculty roles. Medical educators face professional and emotional impacts. As students increasingly use AI tools in their studies, the faculty’s role shifts from being the main knowledge source to facilitating learning, curating resources, fostering critical thinking, and promoting engagement. This change affects methodology, autonomy, assessment practices, and the broader medical education context.

Across 4 scenarios, each future has a key risk and institutional responsibility. In Analog Happiness, the main risk is missed opportunities because students are emotionally supported but unprepared for clinical practice due to a lack of AI integration. Institutions should include AI literacy in the curriculum, provide ethical guidance, and ensure transparent AI use education.

In Gen AI Paradise, the main challenge is sustainability, not imbalance. Even with systems where innovation and mental health support grow together, efficiency gains can hide psychological strain. Institutions should implement long-term governance, train faculty, and monitor student well-being to maintain this balance over time.

In the “Disconnected Struggles” scenario, the main risk is academic trauma from the lack of mental health support and institutional guidance on generative AI. Students face high expectations, uncertainty, and digital change alone. Action should focus on restoring safety and trust via accessible mental health services and clear rules on ethical AI use.

**Figure 6.**
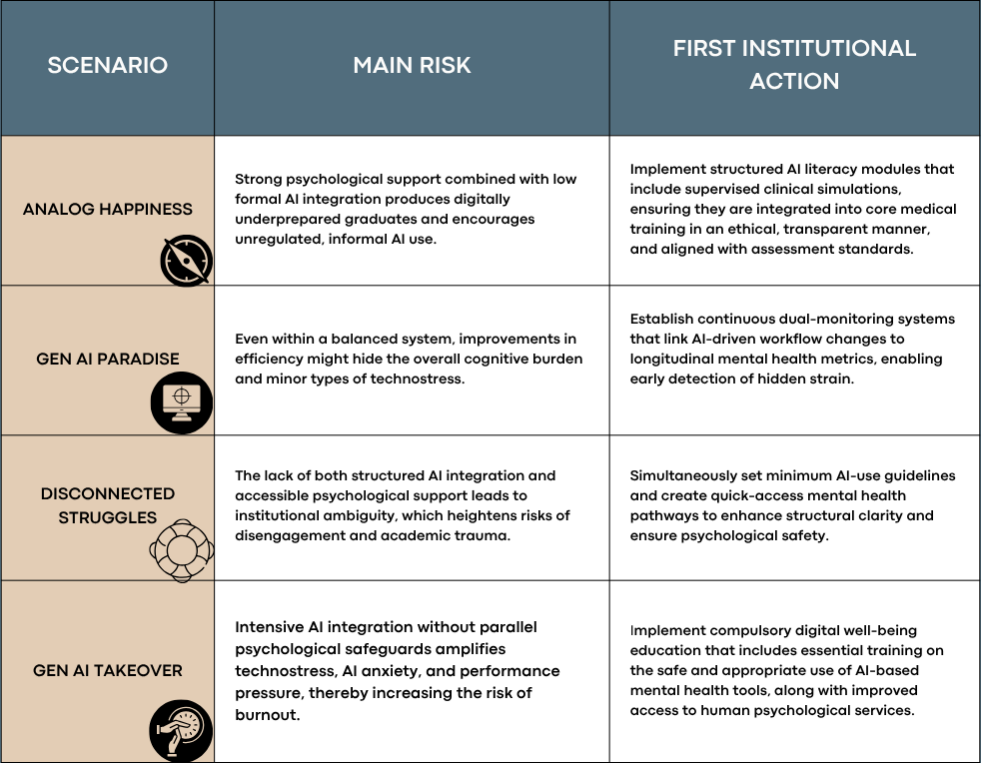
Outline of risks and recommended institutional responses across 4 scenarios in this foresight study. AI: artificial intelligence.

The “Gen AI Takeover” risks creating skilled but emotionally vulnerable graduates as AI’s rapid integration outpaces support systems. This increases technostress, burnout, and performance pressures, threatening learning and clinical practice. Risks can be mitigated by strengthening mental health support and embedding psychological safety into AI-based education, assessment, and feedback. Figure 6 summarizes the main risks of each scenario and provides initial suggestions for institutions as immediate first steps, highlighting key systemic risks associated with generative AI use and the mental health of medical students, along with possible immediate actions for educational institutions. In the visual framework, Analog Happiness is symbolized by a compass indicating direction, “Gen AI Paradise” by a calibration dial indicating balance, “Disconnected Struggles” by a lifebuoy indicating safety, and “Gen AI Takeover” by a brake indicating constraint.

The presence of generative AI also alters the perception of professional authority. When students have instant access to AI-generated answers, the instructor is no longer the sole source of expertise. For some educators, this can feel like a threat, while for others it opens the possibility of highlighting what remains distinctly human in teaching. Such a shift, however, requires careful support, as moving from a position of authority to one of partnership can be experienced as both professionally and personally destabilizing.

Generative AI exposes gaps in digital literacy. Students adopt new tools quickly, but faculty vary in confidence and acceptance. Discrepancies threaten educational quality, highlighting the need for structured training to build teacher competence. The pressure to adapt and learn new skills adds to the psychological burden of academic medicine. Without support, the risk of fatigue and burnout increases. These considerations show that integrating generative AI depends on faculty adaptation. Their ability to adjust will decide if AI improves medical education or worsens inequalities and pressures within the profession [72].

Another area that merits discussion is the future of assessment in medical education. Traditional high-stakes examinations, administered at fixed intervals, may increasingly fail to align with the personalized and dynamic nature of contemporary medical training. Computerized adaptive testing (CAT), grounded in item response theory, offers a promising alternative that has been successfully simulated in national medical assessments. One study using the Standardized Competence Test for Clinical Medicine Undergraduates in China constructed a CAT item bank and found that this approach could significantly reduce test length while maintaining precision and validity. Similarly, psychometric simulations in health professions assessment have shown that CAT can enhance reliability and reduce the burden on the examinee [73,74].

Beyond efficiency, adaptive testing offers competency-based assessment timing. Instead of fixed schedules, systems could test students once they show sufficient mastery. While this could lower stress and match assessments to readiness, it also raises transparency, fairness, and standardization concerns. We also need to acknowledge students’ roles in shaping these systems. Engaged learners in design or evaluation, such as pilot programs, show higher acceptance and trust.

Conversely, lack of involvement can breed skepticism. While direct evidence from medical schools remains limited, broader educational research underscores that student participation can legitimize technology-mediated assessment and ensure it supports pedagogical goals [18,75,76].

Moreover, generative technologies are beginning to automate parts of assessment creation. Generative AI tools have been used to create multiple-choice items tailored to clinical training levels and support test blueprinting and standard setting. Recent comparisons between clinician-written and AI-generated questions are emerging to explore psychometric equivalence and item quality [77].

If assessment becomes more adaptive and technology-mediated, students’ voices should not be an afterthought but a central part of the process. Their involvement can enhance perceptions of fairness, mitigate concerns about automation, and help align assessment systems with actual learning needs. Ultimately, striking a balance between technological capability, institutional responsibility, and student agency will be crucial in ensuring that assessment reform fosters both competence and trust in future physicians.

Generative AI is a fundamental force expected to transform medical education, not just an optional addition. Likewise, mental health support should be seen as essential rather than a luxury, and preparing faculty is as crucial as preparing students. These insights are practical as well: the NHS Topol Review has already highlighted the pressing need to ready the workforce for AI-enabled systems medicine [78], while the CanMEDS 2025 framework explicitly integrates digital health competencies into physician training [79].

At the same time, long-standing practices such as Schwartz Rounds and the Healer’s Art course demonstrate that structured support for emotional well-being can reduce burnout and help students connect to the meaning of their future profession. Finally, global guidance, such as the World Health Organization’s recommendations on AI ethics, underscores that safe integration requires both governance and education. When students feel recognized and supported, they are much more likely to become the kind of clinicians our changing world urgently needs: skilled, compassionate, connected, and profoundly humane. Achieving this requires a collective effort: researchers must speak their truths and highlight what needs to be seen; educators should balance rigor with reverence in shaping minds; students need to claim their right to full support; and decision-makers must invest not only in systems but also in the humanity of future healers.

## Supplementary material

10.2196/85373 Multimedia Appendix 1 Identified phenomena and supporting references.

## Abbreviations

AI: artificial intelligence
CAT: computerized adaptive testing
HC: health care
HCP: health care professional
IMI: internet and mobile-based intervention
LLM: large language model
MS: medical student
SA: scenario analysis
SWOT: Strengths, Weaknesses, Opportunities, Threats
