# Supplementary material for: Generative AI's Impact on the Mental Health of Medical Students: Scenario Analysis
Source: JMIR Med Educ. 2026 May 26;12:e85373. doi: 10.2196/85373 (PMC13211868; doi:10.2196/85373)
Supplement: Multimedia Appendix 1 [file mededu-v12-e85373-s001.docx]

Table S1 summarizes the key phenomena shaping the future of medical education, organized across micro (individual), meso (organizational and institutional), and macro (system-level and societal) levels. The categorization is based on a conceptual framework developed for the scenario analysis. Supporting empirical and theoretical references for each phenomenon are provided below.

| **Level** | **Phenomenon** | **Relevance** | **Literature** |
| --- | --- | --- | --- |
| Macro | The impact of generativeAI, especially ChatGPT | Generative AI tools are already present in higher education, widely used by the masses; their rapid and systemic spread is redefining the basic concepts of education and learning alike | ChatGPT and Generative Artificial Intelligence for Medical Education^1^  Current Status of ChatGPT Use in Medical Education^2^  Integrating Generative Artificial Intelligence into Medical Education^3^ |
| Macro | Post-COVID digital shift | After the pandemic, digital education has not fully returned to its previous form; hybrid and online models have remained in place permanently, bringing new mental challenges with them | Medical Studies during the COVID-19 Pandemic^4^  The impact of COVID-19 on medical education and training^5^   High prevalence of anxiety, depression, and stress among remote online medical students during the COVID-19 pandemic^6^ |
| Macro | The global mental health crisis | According to the WHO and international research, the mental health of young people, especially medical students, is deteriorating, and this trend is observable worldwide. | The role of mental health support for medical students^7^   Supporting the future of medicine: Student mental health services in medical school^8^   Well-being and burnout in medical students: challenges and solutions^9^ |
| Macro | National and international AI-related regulations | While AI regulation regarding ethics and transparency takes place at a macro level, it also has a direct impact on the learning environment.. | Legal and Ethical Consideration in Artificial Intelligence in Healthcare.Who Takes Responsibility?  ^10^  FUTURE-AI: International consensus guideline for trustworthy and deployable artificial intelligence in he  heathcare  ^11^  Legal and Ethical Considerations of Using Artificial Intelligence in Psychiatric Wards^12^ |
| Macro | Characteristics of generation Z | Today’s students learn differently; they have different expectations and competencies, which require systemic adaptation. | Learning styles, preferences and needs of generation Z healthcare students: Scoping review ^13^  Medical student needs for e-learning: perspectives of the generation Z^14^  Applying simulation learning theory to identify instructional strategies for Generation Z emergency medicine residency education^15^ |

| **Level** | **Phenomenon** | **Relevance** | **Literature** |
| --- | --- | --- | --- |
| Meso | Integrating AI into the curriculum | An increasing number of higher education institutions are attempting to integrate AI, but there is still no unified or well-developed methodology for how this should be done, done well. | A scoping review of artificial intelligence in medical education: BEME Guide No. 84 ^16^  Artificial Intelligence Education Programs for Health Care Professionals: Scoping Review^17^  Applications of Artificial Intelligence in Medical Education:A Scoping Review^18^ |
| Meso | Educators’ attitudes towards AI | The reaction of instructors-who are themselves medical doctors-is an important factor in shaping how students will relate to AI. | Perceptions of Faculty and Students About Use of Artificial Intelligence in Medical Education:A Qualitative Study^19^  Decoding medical educators' perceptions on generative artificial intelligence in medical education^20^  Perceptions of Artificial Intelligence Among Healthcare Staff: A qualitative survey study^21^ |
| Meso | Mental health support systems | At many medical faculties, mental health support is either unavailable or insufficient; the systems are overburdened, leaving students to rely on individual coping strategies, all while technostress continues to intensify. | Utilization of counseling services at one medical school^22^   Supporting the future of medicine:Student mental health services in medical school ^8^  Increasing utilization of student mental health services on a college campus. Eight actionable tips^23^ |
| Meso | Assessment and examination methods in the new environment | Generative AI calls into question the validity of traditional forms of assessment, such as tests or essay writing. Students often don’t even know what is allowed and what isn’t, which can lead to additional anxiety. | Should oral examination be reimagined in the era of AI?^24^  Proximity to Practice: The Role of Technology in the Next Era of Assessment ^25^  Applications of Artificial Intelligence for Non-Psychomotor Skills Training in Health Professions Education: A Scoping Review^26^ |
| Meso | Disparities in digital acces | Not all students have the same level of access to devices, digital skills, or even language proficiency—though the latter may be less of a factor in medical schools. It can also be a challenge who can afford paid versions and who must rely on free ones. This may create new forms of inequality within education. | Addressing Digital Divide through Digital Literacy Training Programs: A Systematic Literature Review ^27^ Disparities in Health Care and the Digital Divide^28^ Bridging the digital divide: Promoting equal access to online learning for health professions in an unequal world^29^ |

| **Level** | **Phenomenon** | **Relevance** | **Literature** |
| --- | --- | --- | --- |
| Micro | Individual generative AI usage habits | **Students are using these learning-support tools on a large scale, but many are unaware of the ethical or educational psychology implications of their use.** | Exploring artificial intelligence literacy and the use of ChatGPT and Copilot in academic nursing report writing^30^   Medical students' AI literacy and attitudes towards AI: a cross-sectional two-center study using pre-validated assessment instruments ^31^  Medical students' perceptions of an artificial intelligence (AI) assisted diagnosing program ^32^ |
| Micro | AI-anxiety and technostress | Uncertainty about whether they’re using AI correctly, whether others are ahead, or whether they’re even allowed to use it to such an extent can lead to anxiety and a lack of self-confidence, further intensifying competition in an already high-pressure environment | Medical students' AI literacy and attitudes towards AI: a cross-sectional two-center study using pre-validated assessment instruments^31^  From Theory to Practice: Artificial Intelligence (AI) Literacy Course for First-Year Medical Students^33^  Empowering medical students with AI literacy: A curriculum development journey^34^ |
| Micro | A sense of empowerment and regaining controll | On the other hand, using AI can have the opposite effect for many students: it reduces stress, helps structure the learning material, and restores a sense of control over their studies | Integration of ChatGPT Into a Course for Medical Students: Explorative Study on Teaching Scenarios, Students' Perception, and Applications  ^35^  Medical Student Experiences and Perceptions of ChatGPT and Artificial Intelligence: Cross-Sectional Study^36^  Dialogues with artificial intelligence: Exploring medical students' perspectives on ChatGPt^37^ |
| Micro | Burnout and AI-driven information overload | Excessive, 'anything goes' use of AI often doesn’t reduce but rather increases learning-related anxiety, especially during exam periods, and can lead to indecisiveness, mental blocks, and burnout. | Impact of Providing Peer Support on Medical Students' Empathy, Self-Efficacy, and Mental Health Stigma^38^  Barriers to mental health service utilisation among medical students in Saudi Arabia^39^  Mental Health Service Utilization Among Medical Students with a Perceived Need for Help^40^ |
| Micro | Changes in personal learning strategies | AI may encourage more passive and superficial learning habits, with prompting replacing reading, which, in the long run, can weaken independent thinking skills | Effect of a flipped classroom course to foster medical students' AI literacy with a focus on medical imaging: a single group pre-and post-test study^41^  Developing Medical Education Curriculum Reform Strategies to Address the Impact of Generative AI: Qualitative Study^42^  Artificial intelligence and medical education: application in classroom instruction and student assessment using a pharmacology & therapeutics case study^43^ |
| Micro | Peer-to-peer knowledge and AI tips sharing, AI as peer tutor | New, informal patterns of community learning may emerge, which can serve as both sources of support and potential sources of stress. | Medical students and ChatGPT: analyzing attitudes, practices, and potential implications, also academic  perceptions. ^44^  Health profession students' perceptions of ChatGPT in healthcare and education: insights from a mixed-methods study^45^  Is use of ChatGPT cheating? Students of health professions perceptions^46^ |
| Micro | Individual well-being and mental health | Students are overburdened, fearing the new opportunities and the prospect of having to learn yet another skill, or they turn these opportunities to their own advantage. | The role of mental health support for medical students^7^  Support for medical students with mental health problems: A conceptual model^47^  Perspectives on mental health services for medical students at an Ugandan medical school^48^ |

References

1. Boscardin, C. K., Gin, B., Golde, P. B. & Hauer, K. E. ChatGPT and Generative Artificial Intelligence for Medical Education: Potential Impact and Opportunity. *Academic Medicine* **99**, (2024).

2. Tianhui Xu & Huiting Wen. Current Status of ChatGPT Use in Medical Education: Potentials, Challenges, and Strategies. *JMIR* (2024).

3. Triola, M. M. M. & Rodman, A. M. M. Integrating Generative Artificial Intelligence Into Medical Education: Curriculum, Policy, and Governance Strategies. *Academic Medicine* (2025).

4. Zis, P., Artemiadis, A., Bargiotas, P., Nteveros, A. & Hadjigeorgiou, G. M. Medical studies during the COVID-19 pandemic: The impact of digital learning on medical students’ Burnout and mental health. *Int J Environ Res Public Health* **18**, (2021).

5. Diokno, A. C. & Devries, J. M. The impact of COVID-19 on urologic practice, medical education, and training. *International Urology and Nephrology* vol. 52 Preprint at https://doi.org/10.1007/s11255-020-02511-0 (2020).

6. Xu, T. & Wang, H. High prevalence of anxiety, depression, and stress among remote learning students during the COVID-19 pandemic: Evidence from a meta-analysis. *Frontiers in Psychology* vol. 13 Preprint at https://doi.org/10.3389/fpsyg.2022.1103925 (2023).

7. Gács, B., Tényi, T., Pálfi, K., Major, J. & Horváth-Sarródi, A. The role of mental health support for medical students. Experiences at the University of Pécs. *Orv Hetil* **164**, (2023).

8. Hale, E. W. & Davis, R. A. Supporting the future of medicine: Student mental health services in medical school. *Frontiers in Health Services* **3**, (2023).

9. Bhugra, D. & Molodynski, A. Well-being and burnout in medical students: challenges and solutions. *Ir J Psychol Med* (2022) doi:10.1017/ipm.2022.26.

10. Čartolovni, A., Tomičić, A. & Lazić Mosler, E. Ethical, legal, and social considerations of AI-based medical decision-support tools: A scoping review. *International Journal of Medical Informatics* vol. 161 Preprint at https://doi.org/10.1016/j.ijmedinf.2022.104738 (2022).

11. Karim Lekadir & Alejandro F Frangi. FUTURE-AI: international consensus guideline for trustworthy and deployable artificial intelligence in healthcare. *BMJ* (2025).

12. Barry Solaiman & Abeer Malik. Monitoring Mental Health: Legal and Ethical Considerations of Using Artificial Intelligence in Psychiatric Wards - ADDENDUM. *Am J Law Med .* (2024).

13. Shorey, S., Chan, V., Rajendran, P. & Ang, E. Learning styles, preferences and needs of generation Z healthcare students: Scoping review. *Nurse Education in Practice* vol. 57 Preprint at https://doi.org/10.1016/j.nepr.2021.103247 (2021).

14. Kyong-Jee Kim. Medical student needs for e-learning: perspectives of the generation Z. *Korean med ed* (2024).

15. Michael Hrdy  1 & Emily M Tarver. Applying simulation learning theory to identify instructional strategies for Generation Z emergency medicine residency education. *AEM Educ Train* (2024).

16. Gordon, M. *et al.* A scoping review of artificial intelligence in medical education: BEME Guide No. 84. *Medical Teacher* vol. 46 Preprint at https://doi.org/10.1080/0142159X.2024.2314198 (2024).

17. Charow, R. *et al.* Artificial Intelligence Education Programs for Health Care Professionals: Scoping Review. *JMIR Medical Education* vol. 7 Preprint at https://doi.org/10.2196/31043 (2021).

18. Nagi, F. *et al.* Applications of Artificial Intelligence (AI) in Medical Education: A Scoping Review. in *Studies in Health Technology and Informatics* vol. 305 (2023).

19. Sarah M Salih. Perceptions of Faculty and Students About Use of Artificial Intelligence in Medical Education: A Qualitative Study. *Cureus* (2024).

20. Jorge Cervantes & Blake Smith. Decoding medical educators’ perceptions on generative artificial intelligence in medical education. *J Invwsting Med* (2024).

21. Castagno, S. & Khalifa, M. Perceptions of Artificial Intelligence Among Healthcare Staff: A Qualitative Survey Study. *Front Artif Intell* **3**, (2020).

22. Chang, E., Eddins-Folensbee, F., Porter, B. & Coverdale, J. Utilization of counseling services at one medical school. *South Med J* **106**, (2013).

23. Davis, R. A., Wolfe, J. & Heiman, N. Increasing utilization of student mental health services on a college campus: Eight actionable tips. *Journal of American College Health* **71**, (2023).

24. Prashanti Eachempati & Ramnarayan Komattil. Should oral examination be reimagined in the era of AI? *adv Physiol Educ* (2025).

25. Andrew E Krumm & Hollis Lai  2. Proximity to Practice: The Role of Technology in the Next Era of Assessment. (2024).

26. Kenya A Costa-Dookhan & Zachary Adirim. Applications of Artificial Intelligence for Non-Psychomotor Skills Training in Health Professions Education: A Scoping Reviewhttps://pubmed.ncbi.nlm.nih.gov/39874445/. *Acad Med* (2025).

27. Choudhary, H. & Bansal, N. Addressing Digital Divide through Digital Literacy Training Programs: A Systematic Literature Review. *Digital Education Review* (2022) doi:10.1344/DER.2022.41.224-248.

28. Saeed, S. A. & Masters, R. M. R. Disparities in Health Care and the Digital Divide. *Current Psychiatry Reports* vol. 23 Preprint at https://doi.org/10.1007/s11920-021-01274-4 (2021).

29. Siew Ping Han & Ben Kumwenda  2. Bridging the digital divide: Promoting equal access to online learning for health professions in an unequal world. *Med Educ* (2025).

30. Li-Ping Tseng & Li-Ping Huang. Exploring artificial intelligence literacy and the use of ChatGPT and copilot in instruction on nursing academic report writing. *Nurse Educ Today .* (2025).

31. Matthias Carl Laupichler & Alexandra Aster. Medical students’ AI literacy and attitudes towards AI: a cross-sectional two-center study using pre-validated assessment instruments. *2024*.

32. Robleto, E. *et al.* Medical students’ perceptions of an artificial intelligence (AI) assisted diagnosing program. *Med Teach* **46**, (2024).

33. Max C Anderson & Hunter Levingston. From Theory to Practice: Artificial Intelligence (AI) Literacy Course for First-Year Medical Students. *Cureus* (2024).

34. Ming-Yuan Huang. Empowering medical students with AI literacy: A curriculum development journey. *Med Educ* (2025).

35. Anita V Thomae & Claudia M Witt. Integration of ChatGPT Into a Course for Medical Students: Explorative Study on Teaching Scenarios, Students’ Perception, and Applications. *JMIR Med education* (2024).

36. Alkhaaldi, S. M. I. *et al.* Medical Student Experiences and Perceptions of ChatGPT and Artificial Intelligence: Cross-Sectional Study. *JMIR Med Educ* **9**, (2023).

37. Mehmet Başaran & Cevahir Duman. Dialogues with artificial intelligence: Exploring medical students’ perspectives on ChatGPT . *Med Tech* (2025).

38. Abrams, M. P., Salzman, J., Espina Rey, A. & Daly, K. Impact of Providing Peer Support on Medical Students’ Empathy, Self-Efficacy, and Mental Health Stigma. *Int J Environ Res Public Health* **19**, (2022).

39. Zaenb Alsalman & Marwa Mahmoud Shafey. Barriers to mental health service utilisation among medical students in Saudi Arabia. *Front Public Health* (2024).

40. Phillips, M. S. *et al.* Mental Health Service Utilization Among Medical Students with a Perceived Need for Care. *Academic Psychiatry* **46**, (2022).

41. Laupichler, M. C. *et al.* Effect of a flipped classroom course to foster medical students’ AI literacy with a focus on medical imaging: a single group pre-and post-test study. *BMC Med Educ* **22**, (2022).

42. Shimizu, I. *et al.* Developing Medical Education Curriculum Reform Strategies to Address the Impact of Generative AI: Qualitative Study. *JMIR Med Educ* **9**, (2023).

43. Kannan Sridharan. Artificial intelligence and medical education: application in classroom instruction and student assessment using a pharmacology & therapeutics case study. *BMC Med Educ .* (2024).

44. Ahmed Samir Abdelhafiz. Medical students and ChatGPT: analyzing attitudes, practices, and academic perceptions. *BMC Med Education* (2025).

45. Lior Moskovich. Health profession students’ perceptions of ChatGPT in healthcare and education: insights from a mixed-methods study. *BMC MED Educ* (2025).

46. Abby Swanson Kazley. Is use of ChatGPT cheating? Students of health professions perceptions. *Med Teach .* (2025).

47. Grant, A., Rix, A., Winter, P., Mattick, K. & Jones, D. Support for medical students with mental health problems: A conceptual model. *Academic Psychiatry* **39**, (2015).

48. Kihumuro, R. B. *et al.* Perspectives on mental health services for medical students at a Ugandan medical school. *BMC Med Educ* **22**, (2022).
